# The Controversial Role of Food Allergy in Infantile Colic: Evidence and Clinical Management

**DOI:** 10.3390/nu7032015

**Published:** 2015-03-19

**Authors:** Rita Nocerino, Vincenza Pezzella, Linda Cosenza, Antonio Amoroso, Carmen Di Scala, Francesco Amato, Giuseppe Iacono, Roberto Berni Canani

**Affiliations:** 1 Department of Translational Medical Science and European Laboratory for the Investigation of Food Induced Diseases, CEINGE—Advanced Biotechnologies University of Naples “Federico II”, 80131 Naples, Italy; E-Mails: ritanocerino@alice.it (R.N.); cinzia.pezzella@gmail.com (V.P.); lindacosenza@libero.it (L.C.); antonioamoroso87@gmail.com (A.A.); carmendiscala@gmail.com (C.D.S.); rnocerino13@gmail.com (F.A.); 2 UOC of Gastroenterology “Arnas Civico”, 90127 Palermo post code, Italy; E-Mail: cinzia3006@gmail.com

**Keywords:** cow’s milk allergy, oral food challenge, skin prick test, atopy patch test, hypoallergenic formulas, intestinal microbiota, nutrition

## Abstract

Food allergies (FAs) are an increasing problem in Western countries, affecting up to 10% of young children. FAs are frequently associated with gastrointestinal manifestations. The role of FAs as a potential causative factor for infantile colic (IC) is still controversial. We report the most recent evidence on the pathogenesis, clinical and diagnostic aspects of FA-induced infantile colic (IC) and suggest a stepwise diagnostic approach. We selected articles on clinical and immunologic features, pathogenesis and management of FAs and IC from of 1981 to 2015. Original and review articles were identified through selective searches performed on PubMed, using the following terms: colic, infantile colic, food allergy and infantile colic, infantile colic treatment. The possible relationship between FAs and IC derives from the presence of dysmotility with visceral hypersensitivity and dysbiosis, demonstrated in both conditions, and the clinical response to dietary interventions. Unfortunately, the design of the studies, poor characterization of atopy and different dietary approaches limit the understanding of the importance of FAs in subjects with IC. The role of FAs in IC subjects without other symptoms of atopy remains controversial. However, where there is a suspicion of FAs, a short trial with an extensively hydrolyzed cow’s proteins formula or, if breast fed, with maternal elimination diet may be considered a reasonable option.

## 1. Introduction

Food allergies (FAs) are an increasing problem in Western countries. The overall pooled point prevalence of FAs reported by parents in children aged 0–17 years is 6.86% (95% CI 6.58–7.15) [1]. In young children, the prevalence of FAs has reached 10% [2]. Cow’s milk allergy (CMA) is one of the most common FAs in early childhood. The last 15 years of European prospective cohort studies suggest that the prevalence of CMA is between 1.9% and 4.9%. These results are consistent with a 2002 meta-analysis of 229 articles on FAs: CMA is the most common FA in early childhood with an incidence from 2% to 3% in the first year of life [3]. FAs can cause different gastrointestinal signs and symptoms [4]. It has been proposed that FAs can occur through cutaneous and/or airway sensitization [5,6] Infantile colic (IC), defined according to Rome III criteria as episodes of irritability, fussing or crying that begin and end for no apparent reason and last ≥3h per day, ≥3days per week, for ≥1week [7], is a common condition in the first three months of life, affecting up to about 25% of infants [8]. Even though it mostly resolves by 3–4 months of age, it is often a frustrating problem for parents. It is a frequent cause of medical consultation and is associated with high levels of parental stress and anxiety [8,9]. Despite decades of research, the cause of IC remains partly unknown. FAs have been advocated as a possible cause of IC [10,11]. The possible relationship between FAs and IC derives from two lines of evidence: the presence of dysmotility with visceral neuronal hypersensitivity and dysbiosis demonstrated in both conditions [12,13,14,15,16]; and the clinical response to dietary interventions [17,18,19,20]. In this review, we discuss the most recent evidence on the pathogenesis, clinical and diagnostic aspects of IC, investigating a potential role of FAs. For this purpose, we performed a selective search of original and review articles on PubMed using the following terms: colic, infantile colic, food allergy and infantile colic, infantile colic treatment. We propose a stepwise diagnostic approach in order to limit unnecessary dietary procedures, thereby resulting in a better management of this condition.

## 2. Evidence

### 2.1. Dysmotility and Dysbiosis

**Figure 1:**
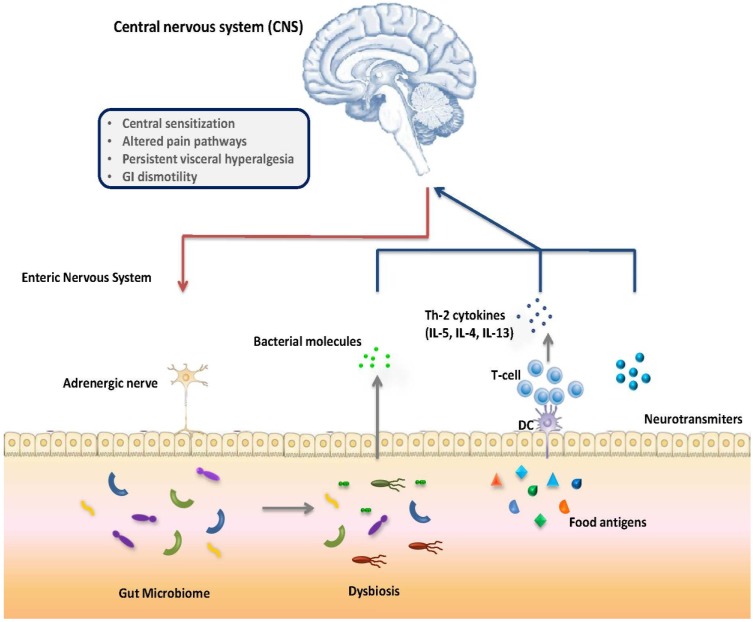
Gut-brain axis interactions in food allergy-induced infantile colic.

Although the etiology of IC remains controversial, dysmotility and gut neuronal hyperexcitability have been advocated as pivotal pathogenetic factors (Figure 1). Similarly, dysmotility and gut neuronal hyperexcitability have been demonstrated in subjects with FA [12,13,14,15,16,21]. Gastrointestinal peristalsis is controlled by a complex neuronal network, the enteric nervous system (ENS). There is evidence of gut dysmotility associated with increased inflammatory cytokine expression in subjects affected by FA [15,22]. The release of mast cell mediators, T helper 2-type cytokines and other neurotoxic compounds, resulting from FA reaction, affect the ENS and cause dysmotility [14]. Experimental studies implicate T helper 2 cells and the cytokines, interleukin (IL)-4 and IL-13, in antigen-induced dysmotility, and IL-5 in the pathogenesis of mucosal eosinophilia. Both mast cells and eosinophils play obligatory roles in different forms of experimental antigen-induced dysmotility. Overall clinical findings appear to implicate eosinophil infiltration in proximal and distal gut dysmotility (esophageal, gastric and colorectal) and mast cell degranulation in mid-gut dysmotility [13]. In a mouse model of FA, a decreased response to muscarinic agonist associated with increased levels of pro-inflammatory cytokines, such as IL-6 and IL-4, in jejunum after allergen exposure has been demonstrated [13]. Histological findings associated with CMA include the presence of cellular infiltrates and marked increase in eosinophils in the mucosa and submucosa with the involvement of even deeper muscular layers in some cases [23]. Eosinophils activation and degranulation may result in acute and long-lasting effects [24]. A potential mechanistic role for eotaxin and eosinophils in gut dysmotility has been suggested in prior studies, which showed eosinophils in the vicinity of damaged axons in mice exposed to food antigens [13]. The close proximity of eosinophils and epithelial cells was found in tissues affected by allergic gastroenteropathy. Eosinophil granule major basic protein (MBP) alters epithelial colonic barrier function. FA-induced gut neuronal hyperexcitability may lead to altered perception of physiological stimuli, such as intestinal distention and peristalsis, being perceived as painful events [13,25,26]. Moreover, dysmotility and gut neuronal hyperexcitability are some of the proposed mechanisms of functional gastrointestinal disorders (FGIDs). 

The microbiome-gut-brain axis comprises a number of fundamental elements, including the central nervous system (CNS), the neuroendocrine and neuroimmune systems, both the sympathetic and parasympathetic limbs of the autonomic nervous system, the enteric nervous system (ENS) and the gut microbiome [27,28]. Signaling along the axis is facilitated by a complex reflex network of afferent fibers projecting to integrative cortical CNS structures and efferent projections to the smooth muscle in the intestinal wall [29]. Thus, a triad of neural, hormonal and immunological lines of communication combine to allow the brain to regulate several gut functions, including visceral sensitivity, motility, secretion, absorption, local immune defense and food intake [29,30,31]. These connections allow the gastrointestinal tract to modulate brain function [32,33]. Thus, early-life inflammation, such as FAA, may cause changes in the brain-gut axis that ultimately result in central sensitization, altered pain pathways and persistent visceral hyperalgesia, as demonstrated in other preclinical approaches [34,35,36]. 

Figure 1 shows that gastrointestinal peristalsis is controlled by a complex neuronal network, the enteric nervous system (ENS). The first few months of life constitute a vulnerable period for the intestinal colonization by bacteria, important for the development of the immune system. Gut microbiota drives immune system maturation and tolerance acquisition and the development and function of ENS. Early-life inflammation, such as cow’s milk allergy (CMA), is characterized by increased production of proinflammatory cytokines and neurotoxic compounds. As a result, CMA reactions affect the ENS and cause peristaltic dysfunction. Hypersensitivity may lead to altered perception of physiological stimuli, such as intestinal distention and peristalsis, being perceived as painful events. 

Gut microbiota drives immune system maturation and tolerance acquisition, as well as ENS development and function [27,28]. Early epidemiological studies have supported the idea that environment-induced alterations in gut microbiota composition, including mode of delivery, drug exposure, nutrition, stress, degree of hygiene and infections, play a central role in the development of allergic diseases [8]. Previous studies suggest the presence of gut dysbiosis in infants affected by FAs [15,37,38,39]. Similarly, an aberrant gut microbiota has been proposed to affect gut motor function and gas production, which lead to colicky behavior [40]. The increased presence of hydrogen gas produced by anaerobic Gram-negative bacteria and lower counts and specific colonization patterns of intestinal lactobacilli have been found in IC [8]. Coliform bacteria, particularly *E. coli*, were found to be more abundant in the feces of colicky infants, suggesting a role for coliform gut fermentation and consequent excessive air production, aerophagia and pain, typical of IC [41]. Rhoads *et al.* demonstrated elevated levels of fecal calprotectin and higher levels of *Klebsiella* in these subjects, further supporting a role of microbiota in IC pathogenesis [41]. There is evidence that supplementation with the probiotic, *Lactobacillus reuteri* ATCC 55730, and its derivate, *Lactobacillus reuteri* DSM 17938, improves colicky symptoms in infants compared to simethicone and placebo treatment, inducing changes in fecal microbiota, particularly *E. Coli* colonization [41]. 

### 2.2. Dietary Intervention

Several investigators have examined the effect of different dietary interventions on IC. In breastfed infants, hypoallergenic maternal diet may be beneficial for reducing symptoms of colic. A high quality randomized controlled trial reported an absolute risk reduction in infant’s cry/fuss duration when mothers changed from a control diet to a hypoallergenic diet (eliminating dairy foods, eggs, peanuts, tree nuts, wheat, soy and fish) for seven days [16]. On the contrary, elimination of only cow’s milk from the mother’s diet seems to have no effect on the duration of IC in breastfed infants [42]. A variable degree of improvement in symptoms of IC has been observed by using extensively hydrolyzed cow milk proteins formulae (EHF) [43,44]. Forsyth *et al.* alternated standard cow’s milk formula with a casein-based EHF for four days each over a total of 16 days. Total daily crying time increased on standard formula, but decreased on EHF, although the effect diminished after the third switch [45]. Iacono *et al.* in a long-term prospective study have demonstrated that in subjects with severe IC when cow’s milk proteins were eliminated from the diet, there was a remission of symptoms [46]; in the same study population after an 18-month follow-up period, 44% of subjects developed FAs [46].

Conflicting results have been reported for the use of soy-based formula. In a study, 11 of 60 hospitalized colicky infants receiving cow’s milk improved when fed a soy formula [47]. Symptoms did not improve in 32 infants, but disappeared when infants where fed with EHF [47]. On the contrary, Berseth *et al.* found that soy-based formula and partially hydrolyzed formula reduced the symptoms of colic to a similar extent [48]. 

### 2.3. The Management of an Infant with Suspected FA-Induced IC

**Figure 2:**
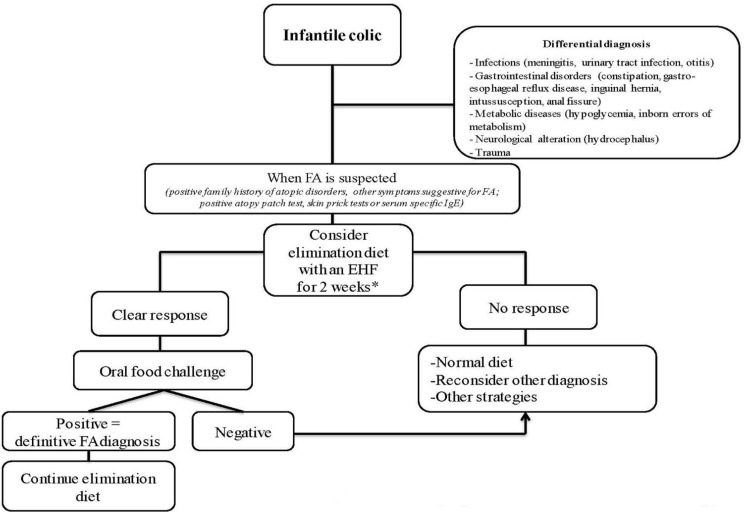
The diagnostic approach for suspected food allergy-induced infantile colic in formula-fed babies. *, If the child is breastfed it could be consider a monitored hypoallergenic maternal diet avoiding eggs, peanuts, tree nuts, wheat, soy, fish, cow’s milk protein containing foods; EHF: extensively hydrolyzed formula; FA: food allergy; IgE: immunoglobulin E.

The concomitant presence of a positive family history for atopic disorders, vomiting, stool pattern modifications or extra-intestinal symptoms of atopy (eczema and/or wheezing) strongly suggests the presence of FA in a subject with IC. In this case, a prompt evaluation of the infant, including in-depth patient history, physical examination, response to elimination diet and to oral food challenge (OFC), is important to confirm a final diagnosis of FAs [49]. FA screening tests (skin prick test, atopy patch test, serum-specific IgE) could be useful, but they are not mandatory for diagnosis [50,51]. Apart from FAs, the differential diagnosis of IC should include: infections, functional constipation, gastro-esophageal reflux disease, inguinal hernia, intussusception, anal fissure, metabolic and neurological alterations and trauma [52]. In order to limit unnecessary dietary procedures, we suggest a stepwise diagnostic approach reported in Figure 2.

### 2.4. Dietary Approach

Appropriate nutritional indications must be provided to ensure primarily an adequate caloric intake, in addition to macro- and micro-nutrients. Furthermore, an appropriate follow-up plan is warranted to assess the compliance to the diet, to identify early signs of nutritional deficiencies and verify the development of tolerance [53,54]. If a baby with IC and no evidence of FAs is thriving on standard formula milk, there is no need to change the feeding strategy. In this case, administration of *Lactobacillus reuteri* DSM 17938 could be considered to reduce daily crying and fussing time [55,56,57,58]. For infants with severe IC and suspected FAs, dietary advice is distinguished according to the type of feeding: in breastfed infants, a monitored hypoallergenic maternal diet avoiding eggs, peanuts, tree nuts, wheat, soy, fish, cow’s milk and cow’s milk protein containing foods is suggested [10,59]; in bottle-fed infants, an empiric time-limited (of about two weeks) therapeutic trial with an extensively hydrolyzed cow milk proteins formula (EHF) could be considered. If a clear clinical response is observed, the trial diet should be continued [10]. If there is no definite benefit after two weeks, the dietary restrictions should be lifted. Soy protein formulae should not be used in subjects aged <6 months, because of the nutritional disadvantages and the content of high concentrations of phytate, aluminum and phytoestrogens (isoflavones), which might have untoward effects [60,61]. In 2008, the American Academy of Pediatrics stated that “the routine use of isolated soy protein-based formula has no proven value in the management of IC or fussiness” [62].

## 3. Conclusions

Food allergy remains over-diagnosed in many Western countries. The most recent available epidemiological data on childhood FAs suggest that the number of true cases of FAs-induced IC should be lower than estimated by physicians. Despite IC and FAs sharing similar pathogenetic aspects, definitive clinical evidence on the true link between these two conditions is still lacking. Different design, poor characterization of atopy in enrolled subjects and different dietary approaches limit our understanding of the impact of FAs in IC. Some authors showed a positive response using partially hydrolyzed formula, which is not recommended for the treatment of CMA [63]. This leads to the hypothesis that modified protein content and osmolarity, rather than a true therapeutic activity on FA, could be responsible for the positive outcome in these cases. In fact, it has been demonstrated that these two factors are able to modulate gut motility [62]. However, despite these limitations and waiting for more data on the FA and IC link, when FAs are suspected in a baby with IC (*i.e*., atopy risk, other signs or symptoms of FA), a short trial with a EHF or, if breast fed, with a maternal hypoallergenic diet (eliminating dairy foods, eggs, peanuts, tree nuts, wheat, soy and fish) may be considered a reasonable option. 
